# Severe Concurrent Hypocalcemia and Hypophosphatemia Following Ferric Carboxymaltose After Roux-en-Y Gastric Bypass: A Comparative Two-Patient Case Series

**DOI:** 10.7759/cureus.117621

**Published:** 2026-10-08

**Authors:** Isra Ali, Ahmed Mahgoub, Anaida Singh, Abu-Bekr Mohamed, Ahmed Elhassan

**Affiliations:** 1 Biomedical Sciences, Mohammed Bin Rashid University of Medicine and Health Sciences, Dubai, ARE; 2 Internal Medicine, Tanta University Hospital, Tanta, EGY; 3 Medicine, St. George's University, St. George's, GRD; 4 Internal Medicine, Detroit Medical Center/Wayne State University, Detroit, USA; 5 Endocrinology, Mohammed Bin Rashid University of Medicine and Health Sciences, Dubai, ARE

**Keywords:** bariatric surgery, ferric carboxymaltose, fibroblast growth factor 23, gastric bypass, hypocalcemia, hypophosphatemia

## Abstract

Ferric carboxymaltose (FCM) can cause hypophosphatemia through fibroblast growth factor 23 (FGF23)-associated renal phosphate wasting. Patients who have undergone Roux-en-Y gastric bypass may be particularly vulnerable because calcium and vitamin D homeostasis may already be impaired. We describe two women with previous Roux-en-Y gastric bypass who developed severe symptomatic concurrent hypocalcemia and hypophosphatemia after FCM. Both had preexisting mineral vulnerability, and the findings are therefore interpreted as a multifactorial interaction rather than evidence that FCM alone caused the hypocalcemia. The first patient, aged 39 years, received FCM 500 mg weekly for five weeks (cumulative dose approximately 2,500 mg) and developed calcium 5.3 mg/dL and phosphate 0.8 mg/dL, with elevated C-terminal FGF23 and inappropriate urinary phosphate excretion during hypophosphatemia. The second patient, aged 37 years, received FCM 500 mg weekly for two doses (cumulative dose 1,000 mg) and presented approximately seven weeks later with calcium 5.9 mg/dL and a subsequent phosphate nadir of 1.3 mg/dL. Her C-terminal FGF23 was measured during early biochemical recovery and remained elevated; urinary phosphate excretion remained measurable during the hypophosphatemic/repletion period, although interpretation was limited by concurrent treatment. Both required prolonged mineral replacement, and the second patient required multiple admissions and repeated IV calcium. These cases suggest that FCM-associated phosphate disturbance may act as an additional metabolic stressor in post-bariatric patients with reduced calcium and vitamin D reserve, producing a severe combined mineral phenotype rather than isolated hypophosphatemia.

## Introduction

Ferric carboxymaltose (FCM) is widely used for iron deficiency when oral iron is ineffective, poorly tolerated, or inadequately absorbed. Hypophosphatemia is a recognized adverse effect of FCM and is mediated principally by increased biologically active fibroblast growth factor 23 (FGF23), which reduces proximal tubular phosphate reabsorption and suppresses 1,25-dihydroxyvitamin D. In randomized physiological studies, this response has been accompanied by reductions in serum phosphate and calcium and increases in parathyroid hormone (PTH) [1,2]. A systematic review and meta-analysis of prospective trials found substantially higher hypophosphatemia rates with FCM than with ferric derisomaltose, with normal kidney function and more severe iron deficiency among factors associated with greater risk [3]. C-terminal FGF23 assays detect both intact FGF23 and C-terminal fragments and therefore do not directly quantify biologically active intact FGF23.

Patients with previous bariatric surgery represent a clinically important risk group because iron deficiency commonly requires parenteral replacement while calcium and vitamin D homeostasis may already be impaired. Bariatric guidelines emphasize long-term surveillance and supplementation for micronutrient deficiencies, including calcium, vitamin D, and iron, after Roux-en-Y gastric bypass [4]. A prospective cohort of post-bariatric patients found new-onset hypophosphatemia in 29% after FCM, accompanied by increased urinary phosphate excretion and increased intact FGF23 [5]. A later retrospective series of patients with Roux-en-Y gastric bypass reported hypophosphatemia in 63.6% after FCM [6]. These observations establish post-bariatric patients as a susceptible population but do not by themselves explain the occurrence of profound concurrent hypocalcemia.

We present two patients with previous Roux-en-Y gastric bypass who had a closely similar phenotype of severe symptomatic hypocalcemia and hypophosphatemia after FCM, accompanied by elevated C-terminal FGF23 and urinary phosphate findings that supported, with differing strength between cases, an FGF23-associated phosphate disturbance. One patient was previously reported individually [7]. She is included here, with explicit citation of the original report, to permit a new comparative analysis with a second independently identified patient; the present manuscript does not reproduce the original text, tables, or figures. The purpose of this comparison is not to attribute the entire mineral disorder to FCM but to examine how FCM-associated phosphate disturbance may interact with preexisting post-bariatric calcium and vitamin D vulnerability.

## Case presentation

Case 1

A 39-year-old woman had undergone Roux-en-Y gastric bypass approximately six years before presentation. Her subsequent history included iron deficiency anemia, vitamin D deficiency, and post-bariatric malabsorption. She initially used calcium and vitamin supplementation after surgery but had discontinued regular supplementation several years before presentation. Because oral iron was not tolerated and parenteral replacement was required, she received FCM 500 mg weekly for five weeks, for a cumulative dose of approximately 2,500 mg.

Following FCM therapy, she presented with generalized muscle cramps and spasms, paresthesia, perioral numbness, blurry vision, fatigue, imbalance, and difficulty walking. Serum calcium was 5.3 mg/dL (reference range 8.9-10.2 mg/dL), phosphate 0.8 mg/dL (2.7-4.5 mg/dL), albumin 3.7 g/dL (3.4-4.8 g/dL), PTH 70 pg/mL (6.2-29 pg/mL), 25-hydroxyvitamin D 19.4 ng/mL (30-100 ng/mL), and magnesium 1.69 mg/dL (1.7-2.55). Electrocardiography showed sinus rhythm with a corrected QT interval (QTc) of 451 ms.

During further evaluation, C-terminal FGF23 was elevated at 156 RU/mL (reference range 26-110 RU/mL). Twenty-four-hour urinary phosphate excretion was 1,316.7 mg/24 h (laboratory reference 400-1,300), an inappropriately high excretion in the presence of marked hypophosphatemia. Twenty-four-hour urinary calcium was reduced to 72.6 mg/24 h (reference range 100-300 mg/24 h). Serum 1,25-dihydroxyvitamin D was 54.4 pg/mL (reference range 19.9-79.3 pg/mL). Paired serum and urine measurements required to calculate fractional phosphate excretion or tubular maximum phosphate reabsorption per glomerular filtration rate (TmP/GFR) were not available.

She required IV calcium and phosphate replacement with magnesium repletion, followed by oral calcium, phosphate, vitamin D3, alfacalcidol, and magnesium. Symptoms and biochemical abnormalities recurred after initial improvement, requiring further hospital treatment. Calcium remained approximately 5.5-8.3 mg/dL and phosphate approximately 1.1-2.6 mg/dL during early follow-up before eventual normalization. Immediately pre-FCM calcium, phosphate, magnesium, PTH, and vitamin D measurements were not reliably available.

Case 2

A 37-year-old woman had undergone laparoscopic Roux-en-Y gastric bypass for class III obesity. During subsequent follow-up, she developed recurrent iron deficiency, vitamin D deficiency, intermittent hypocalcemia, and episodes of hand numbness and lower-limb spasm. She had previously been prescribed calcium and vitamin D but had stopped regular supplementation, and endocrinology documentation described very low dietary calcium intake. Long-term proton-pump inhibitor use was also documented. She had no known renal impairment, previous thyroid or parathyroid surgery, or family history of calcium disorders.

Immediately before the most recent FCM course, she had severe iron deficiency, with ferritin <5 ng/mL and transferrin saturation 2%. PTH was already elevated at 81.5 pg/mL (reference range 6.2-29 pg/mL), supporting preexisting secondary hyperparathyroidism. She had received FCM previously, including prior treatment and a 1,000-mg IV dose. For recurrent iron deficiency, she received FCM 500 mg intravenously weekly for two doses (recent cumulative dose 1,000 mg). One week after completion, serum calcium was 7.8 mg/dL, and 25-hydroxyvitamin D was markedly deficient at 10.6 ng/mL (reference range 30-100 ng/mL).

Approximately seven weeks after completion of the FCM course, she was admitted with dizziness, worsening hand numbness, and carpopedal spasm. Similar symptoms had occurred intermittently since bariatric surgery but had intensified over the preceding two days. Serum calcium reached 5.9 mg/dL (reference range 8.9-10.2 mg/dL), with preserved albumin at 3.9 g/dL. Electrocardiography showed sinus rhythm with QTc 462 ms. PTH remained elevated at 85.2 pg/mL, serum phosphate was 1.7 mg/dL (reference range 2.7-4.5 mg/dL), and 25-hydroxyvitamin D was 18.5 ng/mL. Magnesium transiently fell to 1.32 mg/dL (reference range 1.7-2.55 mg/dL) before normalizing.

She was treated with IV calcium and oral calcium and vitamin D, with initial biochemical and symptomatic improvement, and was discharged after the first admission on oral calcium, a short course of phosphate, and a multivitamin. Symptoms recurred shortly after discharge, with fatigue, palpitations, hand numbness, and lower-extremity spasm. During the recurrent admission, serum phosphate reached a nadir of 1.3 mg/dL (reference range 2.7-4.5 mg/dL). Renal function was preserved during this episode (creatinine 0.4 mg/dL; eGFR 133.1 mL/min/1.73 m²), arguing against impaired renal function as the explanation for the phosphate abnormality.

The subsequent course was prolonged and treatment intensive. Serum calcium repeatedly improved during calcium gluconate infusion and declined again after transition to oral therapy, necessitating additional IV calcium. Oral calcium was changed to calcium citrate and progressively escalated; alfacalcidol, cholecalciferol, phosphate, and magnesium were also administered. During early recovery, 24-hour urinary phosphate excretion was 875.2 mg/24 h (laboratory reference range 400-1,300 mg/24 h), and urinary calcium was 19.2 mg/24 h (reference range 100-300 mg/24 h). Because the collection was obtained after replacement therapy had begun, the phosphate value could not independently establish renal phosphate wasting.

C-terminal FGF23 measured during early biochemical recovery was elevated at 124 RU/mL (reference range 26-110), but the measurement was obtained after the phosphate nadir and after replacement therapy had begun; serum phosphate was 2.7 mg/dL at the time of measurement. Serum 1,25-dihydroxyvitamin D was 33.2 pg/mL (reference range 19.9-79.3). Thus, the FGF23 and urinary phosphate findings were compatible with, but did not directly demonstrate, an FGF23-associated renal phosphate-wasting mechanism in Case 2.

Longer-term follow-up documented multiple admissions requiring IV calcium. Maintenance therapy ultimately included high-dose calcium citrate, alfacalcidol, cholecalciferol, phosphate, and magnesium replacement, with later hydrochlorothiazide. Symptoms and biochemical abnormalities improved gradually, although intermittent facial numbness and mild hypocalcemia persisted during follow-up. The principal clinical and biochemical similarities and differences between the two patients are summarized in Table 1.

**Table 1:** Comparative clinical and biochemical features. Each biochemical result is presented as a single entry, with its reference value in a separate column. Case 1 data are restricted to information reported in the previously published case report [7]. Case 2 C-terminal FGF23 and 24-hour urine measurements were obtained during early recovery after replacement therapy had begun. FCM: ferric carboxymaltose; FGF23: fibroblast growth factor 23; PTH: parathyroid hormone

Parameter	Case 1	Case 2	Reference range
Age	39 years	37 years	Not applicable
Bariatric procedure	Roux-en-Y gastric bypass	Roux-en-Y gastric bypass	Not applicable
Time from bypass to presentation	~6 years	~4 years	Not applicable
Recent FCM cumulative dose	~2,500 mg	1,000 mg	Not applicable
Calcium nadir	5.3 mg/dL	5.9 mg/dL	8.9-10.2 mg/dL
Phosphate nadir	0.8 mg/dL	1.3 mg/dL	2.7-4.5 mg/dL
PTH	70 pg/mL	85.2 pg/mL	6.2-29 pg/mL
25-hydroxyvitamin D at presentation	19.4 ng/mL	18.5 ng/mL	30-100 ng/mL
Magnesium nadir	1.69 mg/dL	1.32 mg/dL	1.7-2.55 mg/dL
C-terminal FGF23	156 RU/mL	124 RU/mL	26-110 RU/mL
24-hour urine phosphate	1,316.7 mg/24 h	875.2 mg/24 h	400-1,300 mg/24 h
24-hour urine calcium	72.6 mg/24 h	19.2 mg/24 h	100-300 mg/24 h
1,25-dihydroxyvitamin D	54.4 pg/mL	33.2 pg/mL	19.9-79.3 pg/mL

The proposed interaction between FCM-associated phosphate wasting and reduced post-bariatric mineral reserve is illustrated in Figure 1.

**Figure 1:**
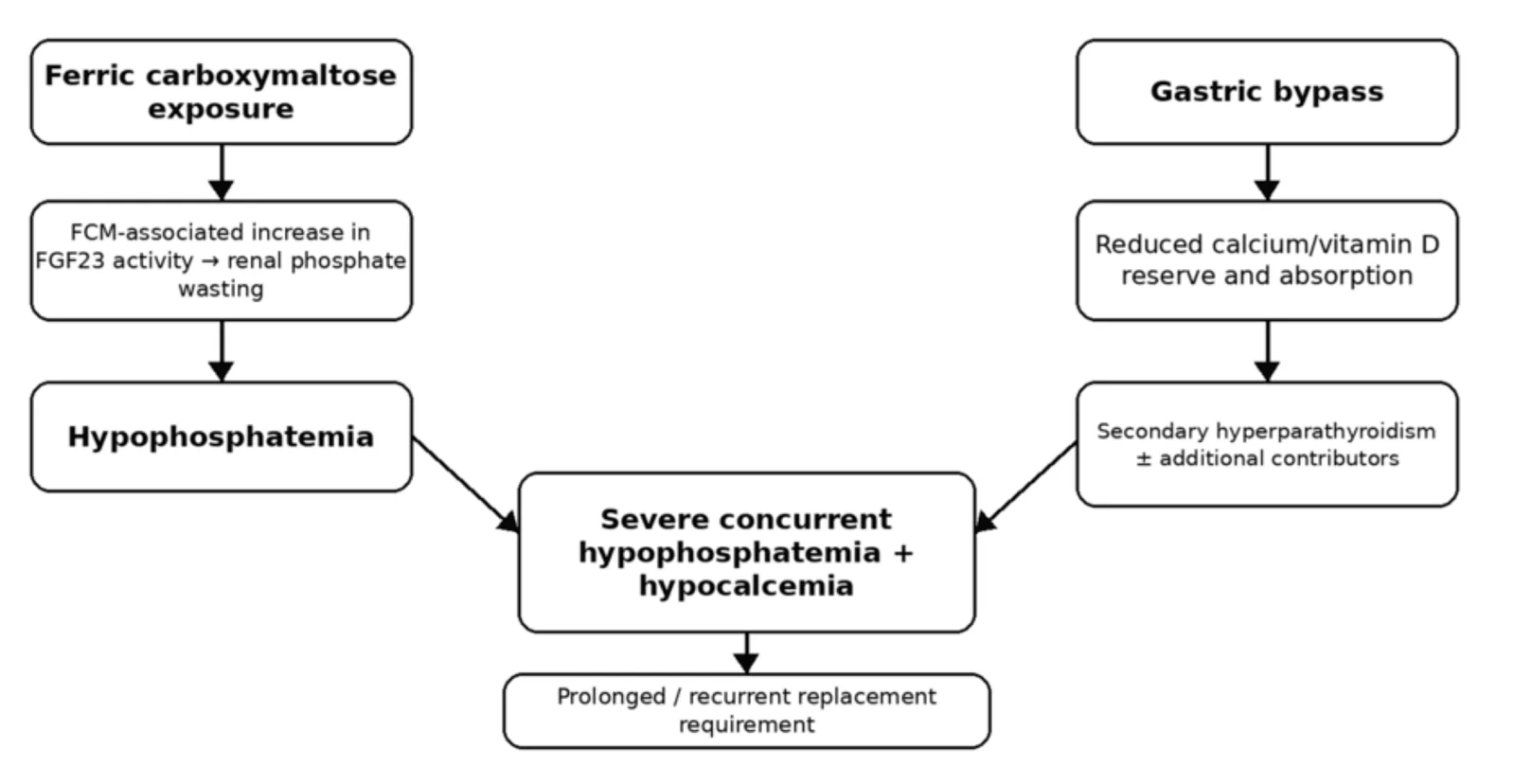
Proposed interaction between FCM-associated phosphate wasting and post-bariatric mineral vulnerability. The diagram is a conceptual model based on established FCM physiology and the clinical interpretation of these cases; it does not imply that every pathway was directly demonstrated in both patients. FCM: ferric carboxymaltose; FGF23: fibroblast growth factor 23

## Discussion

The two patients shared a clinically important phenotype: severe symptomatic hypocalcemia occurring with marked hypophosphatemia after FCM exposure, elevated C-terminal FGF23 concentrations, biochemical findings compatible with renal phosphate loss, and a prolonged requirement for mineral replacement. The comparison is informative because both events occurred on a background of impaired post-bariatric mineral reserve. Case 2 developed a similar phenotype after a lower recent cumulative FCM dose than Case 1; this descriptive difference does not establish a dose-response relationship.

FCM-associated hypophosphatemia is mechanistically linked to increased biologically active FGF23, which reduces proximal tubular phosphate reabsorption and suppresses 1,25-dihydroxyvitamin D. The resulting reduction in intestinal calcium availability can stimulate PTH, which may further increase phosphaturia [1,2]. Patients with previous bariatric surgery are particularly relevant because iron deficiency frequently requires parenteral replacement while calcium and vitamin D homeostasis may already be impaired. In a prospective post-bariatric cohort, 29% developed new-onset hypophosphatemia after FCM, accompanied by increased urinary phosphate excretion, increased intact FGF23, and reduced 1,25-dihydroxyvitamin D [5]. In a retrospective Roux-en-Y gastric bypass series, 63.6% developed hypophosphatemia after FCM [6].

The clinically relevant contribution of this comparison is the severe combined phenotype rather than FCM-associated hypophosphatemia alone. In both patients, hypophosphatemia occurred within a broader mineral disturbance dominated clinically by symptomatic hypocalcemia and prolonged replacement requirements. Case 2 had substantial vulnerability before the most recent FCM course, including intermittent hypocalcemia, very low dietary calcium intake, inconsistent supplementation, long-term proton-pump inhibitor exposure, severe vitamin D deficiency, and elevated PTH. The temporal and biochemical evidence linking FCM to the phosphate disturbance is stronger than that linking FCM directly to the hypocalcemia; the latter was likely multifactorial and substantially influenced by preexisting post-bariatric calcium and vitamin D deficiency. Together, the findings support a model in which FCM-associated phosphate disturbance acted as an additional metabolic stressor superimposed on impaired calcium and vitamin D homeostasis. This background vulnerability is well supported by the bariatric literature: long-term systematic review data show persistent hypovitaminosis D and secondary hyperparathyroidism after Roux-en-Y gastric bypass [8], a later meta-analysis estimated secondary hyperparathyroidism in approximately 39% of patients after RYGB, with prevalence increasing with longer follow-up [9], and a 2023 systematic review and meta-analysis estimated vitamin D deficiency in 42% after RYGB, with higher prevalence at longer follow-up [10].

The strength of evidence for renal phosphate wasting was not identical in the two cases. In Case 1, the published report documented severe hypophosphatemia with elevated C-terminal FGF23 and 24-hour urinary phosphate excretion that was inappropriately high during hypophosphatemia [7]. In Case 2, renal function was preserved during the acute episode, with creatinine 0.4 mg/dL and eGFR 133.1 mL/min/1.73 m². A 24-hour urine phosphate value of 875.2 mg/24 h was obtained during recovery and replacement; because treatment had already begun and paired measurements required for fractional phosphate excretion or TmP/GFR were unavailable, this value could not independently establish renal phosphate wasting. C-terminal FGF23 remained elevated at 124 RU/mL during early recovery. These findings were compatible with an FGF23-associated phosphate disturbance but were less definitive than those in Case 1. In addition, a C-terminal assay does not directly quantify biologically active intact FGF23; accordingly, we avoid claiming that FGF23-mediated phosphate wasting was directly demonstrated in both patients.

The refractory calcium course provides additional clinical evidence that the event was not a transient laboratory abnormality. Case 2 repeatedly improved during IV calcium infusion and deteriorated after transition to oral replacement, resulting in multiple admissions and escalation to high-dose calcium citrate, alfacalcidol, vitamin D, phosphate, and magnesium. Hypomagnesemia may have contributed to the hypocalcemia in both patients, particularly in Case 2, but in Case 2 magnesium normalized while calcium and phosphate abnormalities persisted. The initial QTc of 462 ms also provides objective evidence of clinically important electrolyte disturbance, although a direct causal relationship between the QTc change and calcium replacement cannot be established from these data.

The temporal relationship also warrants caution. Case 2 presented approximately seven weeks after completing the most recent FCM course, although symptoms had been present for approximately one month before admission. This interval is longer than the typical early biochemical nadir reported in many prospective studies, but persistent and delayed-recognition hypophosphatemia after FCM have been described [2,11]. Repeated prior FCM exposure may also have contributed to susceptibility. Nevertheless, the interval, preexisting hypocalcemia, severe vitamin D deficiency, secondary hyperparathyroidism, and other post-bariatric factors preclude attribution of the entire event to FCM.

Recent expert consensus recommends risk assessment before FCM and phosphate monitoring in patients at increased risk or receiving repeated or high-dose courses [12]. Both patients in this report had clinically important risk features, including Roux-en-Y gastric bypass and abnormalities of calcium/vitamin D homeostasis; Case 2 also had repeated prior FCM exposure. Broader evidence reinforces the formulation-specific nature and clinical relevance of this adverse effect: a 2025 systematic review found substantially higher reported hypophosphatemia rates with FCM than with comparator IV iron formulations [13], randomized trials in iron-deficiency anemia reported markedly more hypophosphatemia with FCM than ferric derisomaltose [14], and PHOSPHARE-IBD similarly demonstrated a higher incidence with FCM in patients with inflammatory bowel disease [15]. In high-risk patients, assessment of calcium, phosphate, vitamin D, PTH, and magnesium before treatment may help identify reduced mineral reserve, while post-treatment phosphate and calcium measurements should be guided by symptoms, baseline risk, and repeated dosing. These cases do not define an optimal monitoring schedule.

Several limitations should be acknowledged. This two-patient report is hypothesis-generating and cannot establish incidence or causality, define an optimal monitoring schedule, or show that another IV iron formulation would have prevented the events. One patient was previously published individually [7] and is included here, with explicit citation, for a new comparative analysis; the original text, tables, and figures are not reproduced. Laboratory testing was performed according to clinical need rather than a standardized prospective protocol, and timing differed between cases. Immediate pre-FCM mineral measurements were incomplete in Case 1 as reported in the published case. In Case 2, the C-terminal FGF23 measurement and 24-hour urine collection were obtained during recovery and replacement rather than simultaneously with the phosphate nadir, and paired measurements required for fractional phosphate excretion or TmP/GFR were unavailable. The C-terminal rather than intact FGF23 assay further limits mechanistic inference. Finally, both patients had substantial preexisting post-bariatric mineral vulnerability; therefore, the findings support a multifactorial interaction rather than attribution of the full biochemical phenotype to FCM alone.

Clinically, these cases suggest that the relevant phenotype may extend beyond isolated FCM-associated hypophosphatemia in selected high-risk patients. In this setting, FCM-associated phosphate disturbance may act as an additional stressor superimposed on impaired calcium and vitamin D homeostasis. Management in such patients may therefore warrant attention to the broader mineral phenotype, including calcium, phosphate, vitamin D, magnesium, PTH, dietary calcium intake, and post-bariatric absorption, rather than phosphate replacement alone.

## Conclusions

These two cases illustrate that clinically significant mineral disturbance after FCM may extend beyond isolated hypophosphatemia in patients with previous Roux-en-Y gastric bypass. Both patients had reduced mineral reserve and developed severe concurrent hypocalcemia and hypophosphatemia with a prolonged treatment course. Biochemical findings strongly supported an FGF23-associated phosphate disturbance in Case 1 and were compatible with this mechanism in Case 2, although mechanistic testing in Case 2 was performed during recovery.

The observations support a multifactorial interaction between FCM-associated phosphate disturbance and preexisting post-bariatric calcium and vitamin D abnormalities. In patients with gastric bypass and other risk factors for mineral deficiency, assessment of baseline mineral status and clinically appropriate post-FCM monitoring may facilitate earlier recognition of severe combined abnormalities.
